# Improved deconvolution of very weak confocal signals

**DOI:** 10.12688/f1000research.11773.2

**Published:** 2017-08-07

**Authors:** Kasey J. Day, Patrick J. La Rivière, Talon Chandler, Vytas P. Bindokas, Nicola J. Ferrier, Benjamin S. Glick

**Affiliations:** 1 Department of Molecular Genetics and Cell Biology, University of Chicago, Chicago, IL, 60637, USA; 2 Department of Radiology, University of Chicago, Chicago, IL, 5841, USA; 3 Integrated Light Microscopy Core Facility, University of Chicago, Chicago, IL, 60637, USA; 4 Computation Institute, University of Chicago Mathematics and Computer Science, Argonne National Laboratory, Argonne, IL, 60439, USA

**Keywords:** deconvolution, Gaussian blur, fluorescence microscopy, confocal microscopy, 4D microscopy, signal-to-noise, Huygens

## Abstract

Deconvolution is typically used to sharpen fluorescence images, but when the signal-to-noise ratio is low, the primary benefit is reduced noise and a smoother appearance of the fluorescent structures. 3D time-lapse (4D) confocal image sets can be improved by deconvolution. However, when the confocal signals are very weak, the popular Huygens deconvolution software erases fluorescent structures that are clearly visible in the raw data. We find that this problem can be avoided by prefiltering the optical sections with a Gaussian blur. Analysis of real and simulated data indicates that the Gaussian blur prefilter preserves meaningful signals while enabling removal of background noise. This approach is very simple, and it allows Huygens to be used with 4D imaging conditions that minimize photodamage.

## Introduction

Deconvolution is an established method for sharpening fluorescence images and removing background noise ( Biggs, 2010; Sage *et al*., 2017). The usual input to a deconvolution algorithm is a Z-stack of optical sections generated by widefield or confocal microscopy. Because the benefits of deconvolution are fully realized when the signals are strong, the creators of deconvolution software recommend capturing a large number of photons while keeping the pixel sizes and Z-step intervals relatively small.

 Those conditions are hard to meet with live cell imaging if Z-stacks are being collected at regular intervals to create a 3D time-lapse (4D) data set ( De Mey *et al*., 2008). Intracellular structures are dynamic, so the images need to be taken rapidly. Moreover, the number of captured photons is severely constrained by the need to avoid photodamage to the cells and fluorophores ( Carlton *et al.*, 2010; Pawley, 2006). Such issues are prominent in our 4D confocal microscopy studies of secretory compartments in yeast cells ( Bevis *et al.*, 2002; Losev *et al.*, 2006; Papanikou *et al.*, 2015). We maximize the scan speed, minimize the intensities of the excitation lasers, and set the pixel sizes and Z-step intervals at the traditionally defined Nyquist limit to achieve a tolerable light exposure while ensuring accurate representation of the imaged structures ( Day *et al.*, 2016; Pawley, 2006). The resulting data sets typically comprise thousands of optical sections and have a low signal-to-noise ratio (SNR).

 Even though the sampling characteristics of our 4D data are not ideal for deconvolution, the Huygens deconvolution software from Scientific Volume Imaging (SVI) can facilitate the analysis. A number of other freeware and commercial software packages are also available for deconvolution ( Biggs, 2010; Sage *et al*., 2017), but in our experience, those programs are unsuitable for processing of multi-channel 4D confocal data due to some combination of cumbersome user interfaces, lack of compatibility with relevant file formats, and inadequate noise removal. Huygens is unique in that it readily deconvolves our data sets ( Day *et al*., 2016; Papanikou *et al*., 2015). This software is widely used in the cell biology research community. Importantly, in addition to removing noise, Huygens smooths the uneven shapes and intensities obtained with low-SNR data to generate images that are easy to view and quantify.

 Huygens works well for certain low-SNR fluorescence images, but when the fluorescence signals are very weak, Huygens may perform poorly ( Arigovindan *et al*., 2013). We encountered this problem when imaging low-abundance proteins associated with yeast organelles. In such experiments, a pixel in the signal-containing portion of a confocal section may capture as few as 1–2 photons. To enable deconvolution of images with a very low SNR, Agard and colleagues developed deconvolution software called ER-Decon, which employs a novel regularization method tailored to fluorescence data ( Arigovindan *et al*., 2013). However, ER-Decon has incompletely defined parameters, and it proved to be challenging to use. We therefore sought a method for processing very weak fluorescence signals with Huygens.

## Methods

### Confocal microscopy and image processing

4D imaging of live yeast cells expressing Vps8-GFP and Sec7-mCherry was performed as previously described ( Day *et al.*, 2016) with a Leica SP5 confocal microscope. Briefly, images were collected at the maximum scan speed with a 63x 1.4 NA objective using a voxel size of 80x80x250 nm, a pinhole setting of 1.2 Airy units, and HyD hybrid detectors in photon counting mode. Z-stacks of 28 optical sections were captured at 2 s intervals with the line accumulation (summing) set to either 8x or 1x. Image manipulations other than deconvolution, including 2D and 3D Gaussian blurs, employed 64-bit ImageJ 1.51i ( http://rsbweb.nih.gov/ij/) (RRID: SCR_003070). This software has a sophisticated Gaussian blur algorithm that chooses a suitable kernel based on the user-specified radius (sigma) value. Multi-channel 8-bit confocal 3D time series data were converted to TIFF format, and the TIFF images were converted to 16-bit format, multiplied by 256, and Gaussian blurred where indicated. After deconvolution, the image stacks were average projected and then scaled to provide a quantitatively accurate view of the fluorescent structures ( Hammond & Glick, 2000), and the series of projections was exported to AVI movie format. An online tool was used to convert the movies to MP4 format ( http://video.online-convert.com/convert-to-mp4).

 For labeling the yeast nuclear envelope and peripheral ER membranes, gene replacement was used to tag Hmg1 with GFP. The accompanying pHMG1-GFP.dna SnapGene file ( Supplementary File 1) shows the plasmid used for the strain construction. That file can be opened with SnapGene Viewer ( http://www.snapgene.com/products/snapgene_viewer/). The construction steps can be visualized using History view, and instructions for tagging Hmg1 by gene replacement can be found in the Description Panel. Confocal imaging of yeast cells expressing Hmg1-GFP was performed with a Leica SP8 confocal microscope using the same parameters as for 4D imaging, except that 31 optical sections were captured. A series of 14 optical sections (numbers 13–26) representing approximately 3 μm from the central portions of the cells were processed and average projected as described above.

### Simulation of weakly fluorescent objects

A simulated point-like fluorescent object was generated in a voxel array of XYZ dimensions 200x200x40 with a voxel size of 80x80x250 nm. The fluorescent object was centered along the Z-axis, and was duplicated at an XY spacing of 20 pixels to create an 8x8 array.

The effective confocal point spread function (PSF) was generated by multiplying simulated excitation and emission PSFs, which were produced by the ImageJ plugin PSF Generator ( http://bigwww.epfl.ch/algorithms/psfgenerator/) using the Born & Wolf 3D optical model. This model is appropriate for an object located next to the coverslip. The plugin was used with the following parameters: refractive index = 1.5, numerical aperture = 1.4, voxel size = 80x80x250 nm, excitation wavelength = 488 nm, emission wavelength = 510 nm.

The effective confocal PSF was convolved with the simulated objects using fast Fourier transform-based 3D convolution, and the image values were normalized so that the maximum pixel value corresponded to an average of 1 detected photon. The resulting image stack represented the average detected image, which comprised a total of 17.7 photons per object. Where indicated, random background noise was included by adding a value of 0.01 photons to every voxel in the average detected image. This information was used as input to a Poisson random number generator, yielding a simulated image stack in which each voxel value was drawn from a Poisson distribution whose mean was equal to the corresponding voxel value in the average detected image.

The output was saved in 8-bit TIFF format, and was scaled so that a pixel value of 255 corresponded to 4 photons. Further processing was carried out as for the live cell confocal data, except that Gaussian blurring and/or deconvolution were performed with 8-bit format. The images were then converted to 16-bit format and multiplied by 256 followed by average projection.

To quantify the signal intensity for an object after average projection, ImageJ was used to create a selection of 20x20 pixels centered on the object, and the integrated density was measured. For the deconvolved image, the numbers were multiplied by a correction factor to compensate for scaling of the image by Huygens.

### Deconvolution

Deconvolution with Huygens Essential 15.10 software ( https://svi.nl/HomePage) (RRID: SCR_014237) was performed on an iMac using up to 40 iterations of the Classic Maximum Likelihood Estimation algorithm with a theoretical PSF. Background correction was automatic, except in the case of the simulated confocal Z-stacks with added background noise, for which the background setting was manually adjusted to 0.8. The SNR setting, adjusted empirically to give satisfactory results, was as follows: 4 for the live cell 4D confocal data; 7 for the confocal images of cells with a labeled nuclear envelope; 7 for the simulated confocal Z-stacks with no added background noise; 1 for the simulated confocal Z-stack with added background noise; or 10 for the widefield data. The other parameters used by the Huygens algorithm were configured for either confocal microscopy of live yeast cells ( Day *et al.*, 2016), confocal microscopy of simulated fluorescent objects under the conditions specified during the simulation, or widefield microscopy under the conditions reported for the ER-Decon software ( Arigovindan *et al*., 2013).

The ER-Decon software and associated image data were obtained from the University of California, San Francisco ( http://msg.ucsf.edu/IVE/Download/). Images of fluorescent yeast Zip1 filaments were obtained as part of the ER-Decon package, and were converted to TIFF format using the Bio-Formats Importer plugin for ImageJ ( http://www.openmicroscopy.org/site/support/bio-formats5.1/).

## Results and discussion

**Figure 1.**
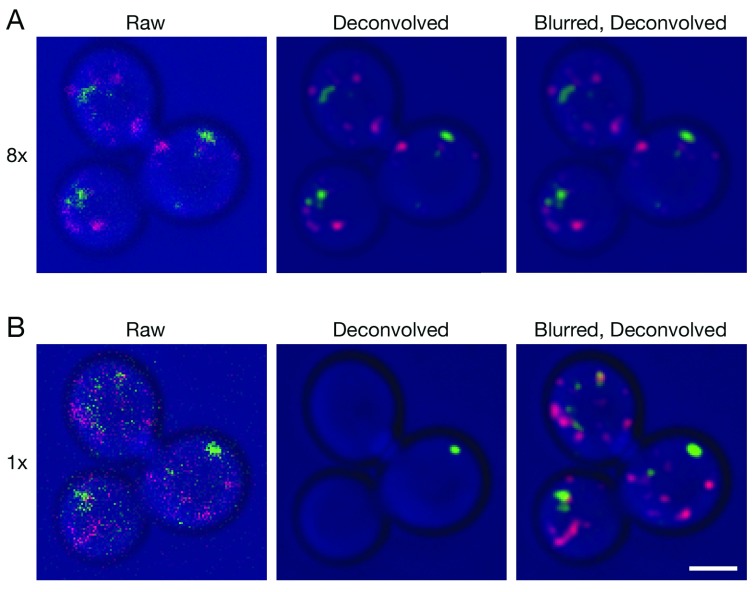
Improved deconvolution of 4D live cell data with a Gaussian blur prefilter. Gene replacement in *Saccharomyces cerevisiae* was used to label late Golgi cisternae with Sec7-mCherry (red) and prevacuolar endosomes with Vps8-GFP (green) ( Papanikou *et al*., 2015). Cells were imaged by 4D confocal microscopy. In consecutive movies, line accumulation was set to ( **A**) 8x or ( **B**) 1x. The data were average projected either with no processing, or after deconvolution with Huygens, or after prefiltering with a 2D Gaussian blur using a radius of 0.75 pixels followed by deconvolution. Fluorescence data are superimposed on differential interference contrast images of the cells (blue). Shown are representative frames from Video 1 (8x) and Video 2 (1x). The fluorescence patterns in ( **A**) and ( **B**) are similar, but not identical because the labeled structures changed during the interval between the two movies. Scale bar, 2 µm.

We generated two small 4D data sets to illustrate confocal imaging of organelles in live *Saccharomyces cerevisiae* cells. The parameters were adjusted to capture either weak signals using a line accumulation of 8x, where each line in the image was scanned eight times and the results were summed ( Video S1), or very weak signals using a line accumulation of 1x, where each line in the image was scanned only once ( Video S2). Projections of representative Z-stacks from the two movies are shown in Figure 1. The organelles were dynamic, so the labeling patterns in the two movies are not identical, but the movies were brief and were captured sequentially, so the labeling patterns are similar enough to allow for comparison. With 8x line accumulation, the raw projections are noisy and display fluorescent structures with uneven shapes and intensities ( Video S1 and Figure 1A). Deconvolution with Huygens efficiently removed the background noise and smoothed the structures. With 1x line accumulation, the data quality is even lower, but fluorescent structures can still be discerned in the raw projections ( Video S2 and Figure 1B). In this case, deconvolution with Huygens erased almost all of the fluorescent structures. The processing employed standard settings in the Huygens software, including deconvolution with the Classic Maximum Likelihood Estimation algorithm. Although a larger percentage of the fluorescent structures in very weak data sets could be preserved by greatly reducing the number of deconvolution iterations or by using different SNR or background settings, the preserved structures often had distorted shapes (not shown). Similar loss or poor preservation of very weak fluorescent structures was seen with the Good’s roughness Maximum Likelihood Estimation algorithm, which is recommended for use with noisy confocal data (not shown). Based on these observations, we have continued to use standard settings in Huygens. Our data sets often lie between the two extremes depicted in Figure 1, and when movies are generated after deconvolution, the fluorescent structures blink because a given structure is erased in some movie frames but not in others (see Video S2). Such movies cannot be productively analyzed.

**Figure 2.**
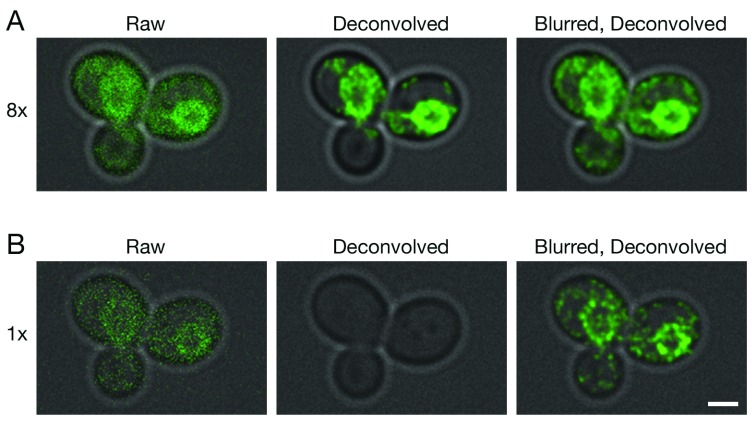
Improved deconvolution of non-punctate fluorescence signals with a Gaussian blur prefilter. Gene replacement in *Saccharomyces cerevisiae* was used to label ER membranes with Hmg1-GFP ( Koning *et al.*, 1996). A confocal Z-stack was captured with line accumulation set to ( **A**) 8x or ( **B**) 1x. The data were average projected either with no processing, or after deconvolution with Huygens, or after prefiltering with a 2D Gaussian blur using a radius of 0.75 pixels followed by deconvolution. Fluorescence data are superimposed on differential interference contrast images of the cells (gray). Scale bar, 2 µm.

**Figure 3.**
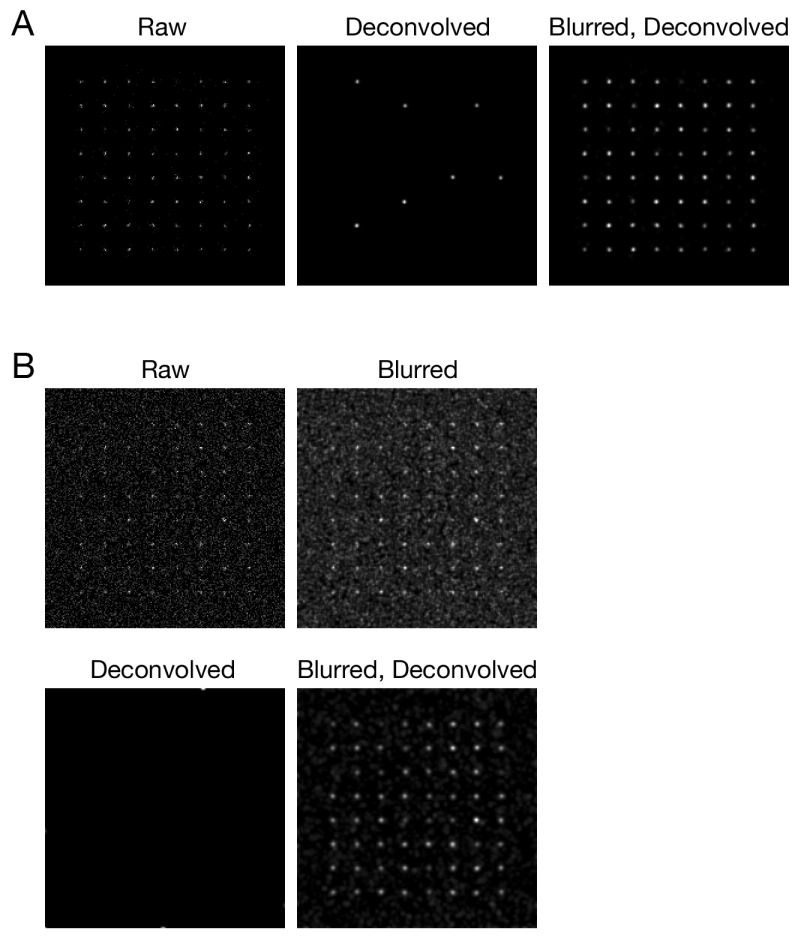
Improved deconvolution of simulated data with a Gaussian blur prefilter. Simulated confocal Z-stacks of fluorescent point sources were created as described in Methods, either ( **A**) without background noise or ( **B**) with background noise. The data were processed and average projected as in Figure 1.

**Figure 4.**
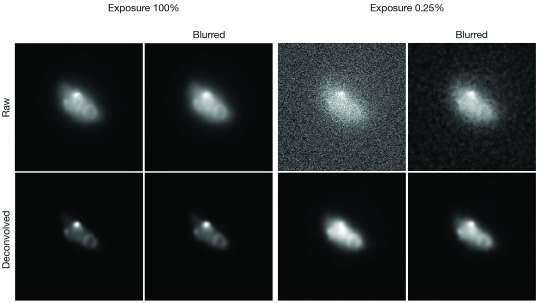
Effect of a Gaussian blur prefilter on deconvolution of widefield fluorescence data. These images of fluorescent yeast Zip1 filaments correspond to Figure 4 of Arigovindan *et al*. (2013). The two exposure levels represent strong (100%) or weak (0.25%) signals, respectively. Where indicated, the data were subjected either to a Gaussian blur with a radius of 1.00 pixel, or to deconvolution with Huygens, or to a Gaussian blur prefilter followed by deconvolution. The theoretical point spread function was based on imaging parameters supplied with ER-Decon.

TIFF files for the experimental and simulated image data are provided in the compressed folder Original Image Files.zip The following files are included: 4D_movie_1x.tif and 4D_movie_8x.tif are the 4D confocal data sets used for Figure 1 and Supplementary Figure 1, and for Video S1 and Video S2; Hmg1_1x.tif and Hmg1_8x.tif are the confocal image stacks used for Figure 2; simulation_80x80x250.tif is the simulated confocal image stack used for Figure 3A and Supplementary Figure 2; simulation_80x80x250_plus_noise.tif is the simulated confocal image stack used for Figure 3B; simulation_40x40x120.tif is the simulated confocal image stack used for Supplementary Figure 3; and Zip1_0.25%.tif and Zip1_100%.tif are the widefield image stacks used for Figure 4. Click here for additional data file. Copyright: © 2017 Day KJ et al. 2017 Data associated with the article are available under the terms of the Creative Commons Zero "No rights reserved" data waiver (CC0 1.0 Public domain dedication).

 In the course of testing several types and combinations of image filters ( Day *et al*., 2016), we discovered that for very weak confocal signals, the key step was to prefilter the optical sections in ImageJ with a Gaussian blur. That prefilter dramatically improved the results obtained after deconvolution ( Video S2 and Figure 1B). Fluorescent structures were no longer erased, and instead were preserved and smoothed while the background noise was largely eliminated. Most of the structures visualized by this method were biologically relevant because they persisted between movie frames ( Video S2). Essentially identical results were obtained with 2D and 3D Gaussian blurs (not shown), so we use a 2D Gaussian blur because the processing is faster. This prefiltering step enables us to generate useful 4D movies from data sets that contain very weak confocal signals.

 Application of the Gaussian blur prefilter requires the data to be in a suitable format. Our images are collected with a high-sensitivity detector in photon counting mode, and the pixel values are in 8-bit format. For very weak signals, typical pixel values are 0, 1, or 2 because a pixel rarely captures more than 2 photons. To obtain a meaningful blur, the numbers are scaled up to allow for intermediate integer values. We convert the images to 16-bit format and multiply by 256, resulting in typical pixel values of 0, 256, and 512. A Gaussian blur then generates a range of intermediate values, effectively spreading the individual photon signals over multiple pixels.

 An important question is how to determine whether a confocal data set is suitable for processing with a Gaussian blur prefilter. Ideally, this prefilter would be used routinely, because even if the average signal intensity is strong, some structures may have very weak signals. The concern with routine application of a Gaussian blur prefilter is that blurring might be propagated to the deconvolved images. Indeed, when the Gaussian blur prefilter was applied to signals strong enough to be preserved during normal deconvolution, we saw some blurring of the fluorescent structures ( Video S1 and Figure 1A). However, this effect was minor with suitable parameters for the prefilter (see below). Our results indicate that a Gaussian blur prefilter can be used to image structures with a range of signal intensities, resulting in preservation of very weak signals without significant degradation of stronger signals.

 Because the labeled structures in our 4D data sets were punctate, we tested whether a Gaussian blur prefilter would also improve deconvolution of other shapes. For this purpose, GFP was fused to a yeast endoplasmic reticulum (ER) protein that localizes mainly to the nuclear envelope ( Koning *et al*., 1996). A single confocal Z-stack was captured at a low excitation laser setting. As shown in Figure 2A, which employed 8x line accumulation, the labeled protein appeared as prominent nuclear envelope rings with weaker labeling of peripheral ER membranes. Deconvolution of the raw data preserved the nuclear envelope rings. When a Gaussian blur was applied before deconvolution, additional signals outside the nuclear envelope were preserved. Figure 2B shows a parallel analysis with 1x line accumulation. In this case, the fluorescence signals were completely erased by deconvolution of the raw data, but application of a Gaussian blur before deconvolution preserved the nuclear envelope rings. We conclude that for various types of fluorescence patterns, a Gaussian blur prefilter preserves very weak confocal signals during deconvolution with Huygens.

 To explore the Gaussian blur effect systematically, and to confirm that it was not limited to the particular configuration of our confocal microscopy setup, we used simulated data. 3D confocal imaging was simulated for an array of 64 faintly fluorescent point-like objects, each of which was represented by about 10–25 photons spread over multiple optical sections. Figure 3A shows projections of this simulated Z-stack before and after processing. After deconvolution with Huygens, only 7 objects were preserved, but after a Gaussian blur prefilter followed by deconvolution, all 64 objects were preserved. The total signal intensities for the objects were largely unchanged after either a Gaussian blur alone or a Gaussian blur followed by deconvolution ( Supplementary Figure 1). A setting of 0.75 pixels for the radius (sigma) parameter of the prefilter preserved signals while causing very little blur in the final images, and similar results were obtained with a radius of 1.00 pixels ( Supplementary Figure 2). We find empirically that radius values of 0.75 – 1.00 pixels work well for both real and simulated fluorescence data obtained under a variety of imaging conditions. When the simulation was repeated with added background noise, a Gaussian blur prefilter followed by deconvolution removed most of the noise while preserving all of the objects ( Figure 3B). In this case, deconvolution in the absence of a prefilter completely erased the objects. The voxels in those simulations were 80x80x250 nm to mimic traditional Nyquist imaging with our confocal system ( Pawley, 2006), but similar results were obtained with voxels of 40x40x120 nm ( Supplementary Figure 3) chosen to meet the more stringent Nyquist criteria recommended by SVI. Thus, a Gaussian blur prefilter preserves weak confocal signals during deconvolution under multiple real and simulated conditions.

 The paper describing the ER-Decon software showed that Huygens could give unsatisfactory results with low-SNR widefield images ( Arigovindan *et al*., 2013). We processed low-SNR widefield microscopy data from that study with a Gaussian blur prefilter before deconvolution. The improvement was only moderate because Huygens did not erase the structures, but when the signal was weak, the prefilter did increase contrast between labeled structures and the background ( Figure 4, right panels), yielding results similar to those obtained with ER-Decon (compare to Figure 4 in Arigovindan *et al.*, 2013). The combined observations demonstrate that a Gaussian blur prefilter consistently improves deconvolution of low-SNR fluorescence data.

 The reason for this beneficial effect of the prefilter is not fully understood. Gaussian blurs suppress high-frequency noise. That approach reduces pixel-to-pixel intensity variations, and it can facilitate analysis methods such as edge detection and particle tracking ( Cheezum *et al*., 2001; Russ & Neal, 2015). A different mechanism presumably underlies the ability of a Gaussian blur to prevent loss of very weak signals during deconvolution. Huygens apparently “expects” a gradually varying distribution of the signal intensities within a set of nearby voxels, and the Gaussian blur prefilter converts the data to a form suitable for the Huygens algorithm.

 Is a Gaussian blur prefilter before deconvolution an acceptable procedure? Processing of images before deconvolution is not generally recommended, but a Gaussian blur is relatively safe. This filter causes a simple and well behaved transformation of the data, and it preserves the total intensity of a fluorescent structure ( Burger & Burge, 2008). Gaussian blurs have previously been employed during deconvolution to suppress noise buildup ( Agard *et al.*, 1989). A Gaussian blur prefilter was actually proposed by the founder of SVI as a method that can reduce noise sensitivity during deconvolution of confocal data ( van Kempen *et al.*, 1997). Therefore, it seems reasonable to apply this prefilter to very weak confocal signals for the novel purpose of avoiding complete erasure of biologically meaningful structures. When the signals are stronger, the Gaussian blur prefilter has a barely detectable effect on the final image, so there seems to be little risk in applying this prefilter routinely.

 It could be argued that the Gaussian blur prefilter merely sidesteps a software flaw, in which case a better option would be to fix the Huygens algorithm. However, Huygens is optimized for processing images that exceed a minimum signal strength, and our confocal data sometimes fall below this threshold. Other deconvolution algorithms may perform differently. The available evidence specifically shows that the Gaussian blur prefilter is useful with Huygens. This straightforward method allows us to take advantage of the flexibility, noise removal capability, and smoothing properties of the Huygens software to process very weak fluorescence signals.

### Update

After seeing the initial version of this manuscript, scientists at SVI analyzed the performance of Huygens with our data sets, and refined their background estimation procedure to improve the deconvolution of images with very weak signals. We find that this change produces excellent results with both real and simulated confocal data obtained under our imaging conditions. The deconvolved images reliably preserve the signals while avoiding the slight loss of sharpness that was observed with a Gaussian blur prefilter. We are grateful to SVI for their responsiveness and constructive feedback.

The improved algorithm is available in Huygens versions 17.04 and later. For investigators running earlier versions of Huygens, the Gaussian blur prefilter remains an effective option for preserving very weak confocal signals during deconvolution. Moreover, the Gaussian blur prefilter may prove to be beneficial for other types of images that are sampled in a manner not well suited to direct processing with Huygens.

## Data availability

The data referenced by this article are under copyright with the following copyright statement: Copyright: © 2017 Day KJ et al.

Data associated with the article are available under the terms of the Creative Commons Zero "No rights reserved" data waiver (CC0 1.0 Public domain dedication). 

**Dataset 1: TIFF files for the experimental and simulated image data are provided in the compressed folder Original Image Files.zip.** The following files are included: 4D_movie_1x.tif and 4D_movie_8x.tif are the 4D confocal data sets used for Figure 1 and Supplementary Figure 1, and for Video S1 and Video S2; Hmg1_1x.tif and Hmg1_8x.tif are the confocal image stacks used for Figure 2; simulation_80x80x250.tif is the simulated confocal image stack used for Figure 3A and Supplementary Figure 2; simulation_80x80x250_plus_noise.tif is the simulated confocal image stack used for Figure 3B; simulation_40x40x120.tif is the simulated confocal image stack used for Supplementary Figure 3; and Zip1_0.25%.tif and Zip1_100%.tif are the widefield image stacks used for Figure 4. doi, 10.5256/f1000research.11773.d163336 ( Day *et al*., 2017).

## Supplementary material

**Supplementary File 1. SnapGene file for the Hmg1-GFP construct.**

**Supplementary Figure 1. Preservation of signal intensities after image processing.** For the simulated array shown in Figure 3A, the intensities after average projection for the eight objects in the top row were measured using either the raw data, or the data after a Gaussian blur with a radius of 0.75 pixels, or the data after a Gaussian blur followed by deconvolution with Huygens. Intensity values are in arbitrary units.

**Supplementary Figure 2. Comparison of different radius values for the Gaussian blur prefilter.** The simulated confocal Z-stack in Figure 3A was subjected to a Gaussian blur prefilter using the indicated radius (sigma) values in pixels, then deconvolved with Huygens and average projected. A radius of 0.50 was not completely effective at preserving the objects, and a radius of 1.00 caused slight blurring in the final image, indicating that a radius of 0.75 was a good compromise.

**Supplementary Figure 3. Effect of a Gaussian blur prefilter with a smaller voxel size.** A simulated confocal Z-stack was generated and processed as in Figure 3A, except that the voxel size was 40x40x120 nm.

**Video S1. Movie generated with weak signals from labeled yeast organelles.** Confocal Z-stacks were collected at 2 s intervals with a line accumulation setting of 8x. The final frame of this movie corresponds to Figure 1A.

**Video S2. Movie generated with very weak signals from labeled yeast organelles.** Confocal Z-stacks were collected at 2 s intervals with a line accumulation setting of 1x. The final frame of this movie corresponds to Figure 1B.
