# Supplementary figures and images for: Improved deconvolution of very weak confocal signals
Source: F1000Res. 2017 Aug 7;6:787. Originally published 2017 Jun 6. [Version 2] doi: 10.12688/f1000research.11773.2 (PMC5553083; doi:10.12688/f1000research.11773.2)

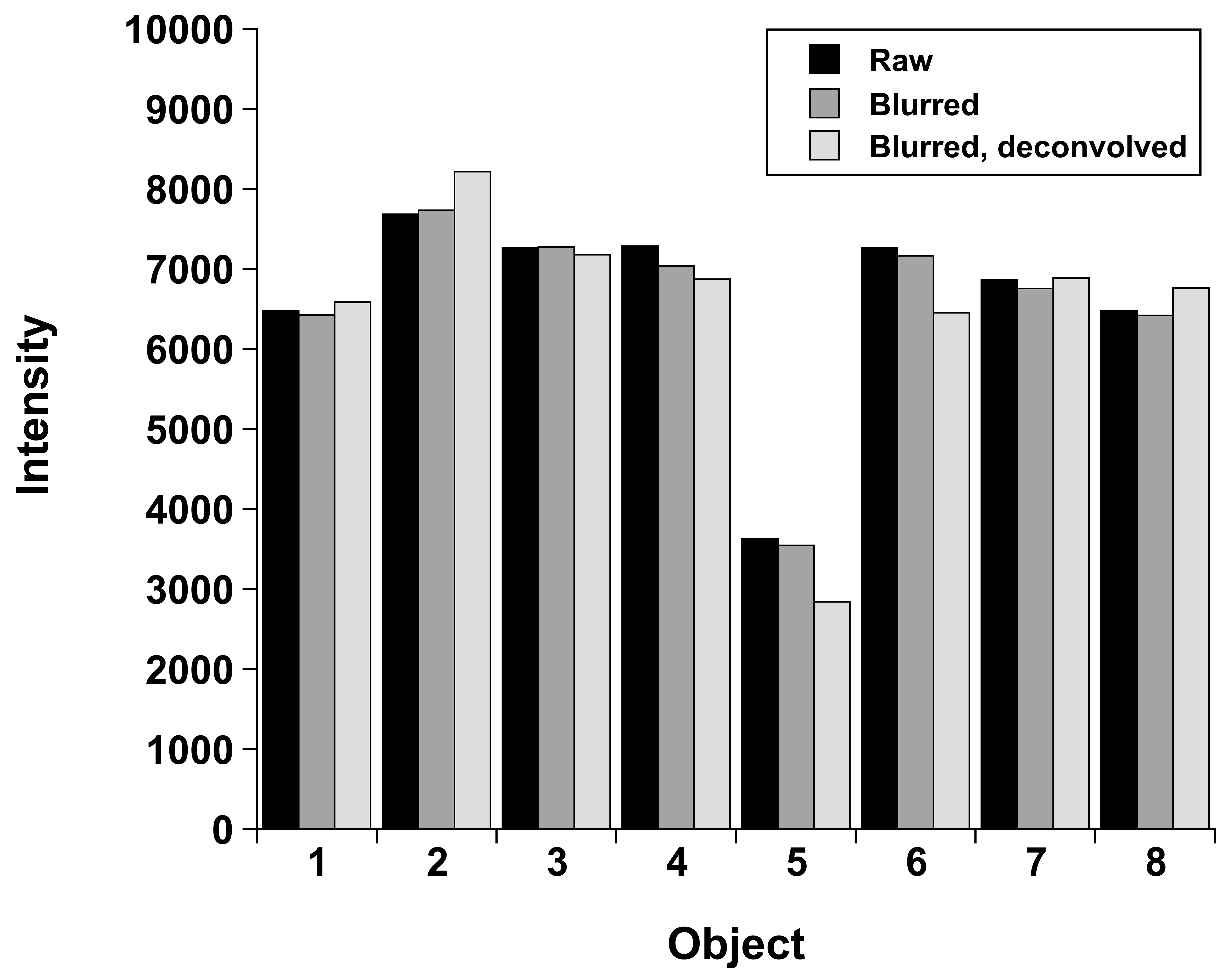

Supplement: Supplementary file 3 [file f1000research-6-13353-s0002.tgz › f291cd33-e87f-47f9-937f-b6b141396671.tif]

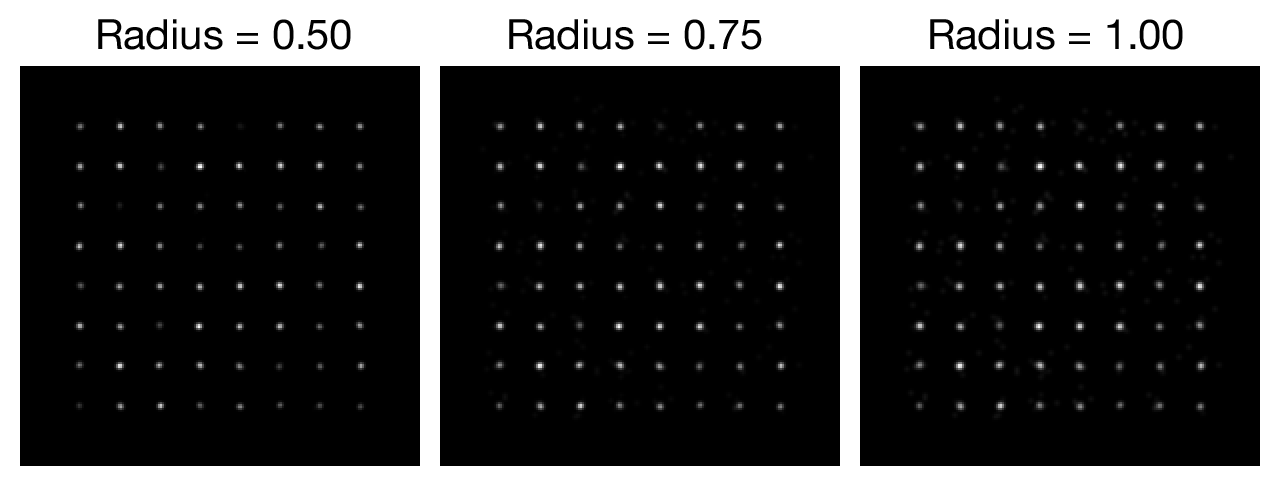

Supplement: Supplementary file 4 [file f1000research-6-13353-s0003.tgz › 452cb8e4-0364-401e-b4da-831399e3f087.tif]

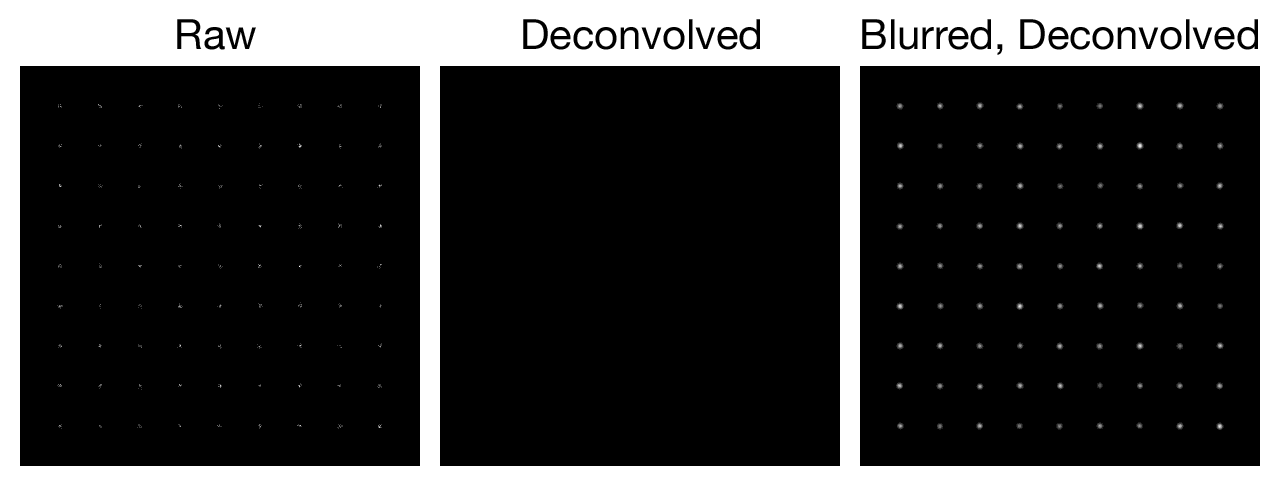

Supplement: Supplementary file 5 [file f1000research-6-13353-s0004.tgz › e8bae752-deee-47e4-99e9-c56b10867ab6.tif]
