# Supplementary material for: Improved deconvolution of very weak confocal signals
Source: F1000Res. 2017 Aug 7;6:787. Originally published 2017 Jun 6. [Version 2] doi: 10.12688/f1000research.11773.2 (PMC5553083; doi:10.12688/f1000research.11773.2)
Supplement: Peer review history

# Peer review: Referee response for version 2

**DOI:** 10.5256/f1000research.13353.r24822

**Published:** 2017-08-08

**Authors:** Akihiko Nakano, Daisuke Miyashiro

**Affiliations:** 1 RIKEN Center for Advanced Photonics, Wako, Japan

It was a kind of surprising outcome, but anyway it was good the SVI adequately addressed the problems. We approve that the authors' revisions are reasonable.

We have read this submission. We believe that we have an appropriate level of expertise to confirm that it is of an acceptable scientific standard.

# Peer review: Referee response for version 1

**DOI:** 10.5256/f1000research.12718.r23779

**Published:** 2017-06-26

**Authors:** Vladimir Denic, Nicholas Weir

**Affiliations:** 1 Department of Molecular and Cell Biology, Northwest Labs, Harvard University, Cambridge, MA, USA; 2 Department of Molecular and Cellular Biology, Harvard University, Cambridge, MA, USA

In this manuscript, Day *et al.* provide a protocol for preventing signal loss resulting from deconvolution of low-intensity fluorescence microscopy images by the Huygens deconvolution software. The authors convincingly demonstrate that image pre-processing using a Gaussian filter prevents loss of low-intensity fluorescent objects in the deconvolved image. Their method is well-described except for the omission of a few details (see below). Their experimental validation demonstrates the power of the method but might be further strengthened by small additional experiments that confirm a one-to-one correspondence between low-intensity structures derived by their image processing and the same structures derived by detection of higher-intensity signals.

 Description of methods:

 The authors clearly describe the rationale for their method and how it fits with pre-existing deconvolution technologies. The flow of their analysis pipeline is generally well-described, though some important details are left out:

1. How is the standard deviation (sigma) of the Gaussian filter determined? Is it empirically optimized to produce minimal loss of signal? After optimization for one image, can the same sigma be reliably applied to other images from the same dataset with consistent results?
2. The authors should address possible artifacts that may arise from spreading fluorescence detected by an SPC to adjacent pixels. Is it appropriate to artificially spread photons from single pixels to adjacent regions that may not contain excited fluorophores? How does this relate point-spread fluorescence of multiple photons to adjacent regions in high-intensity images?

 

 Figures 3-4 contain all essential information for intepretation of the figure and do not require additional work. Minor adjustments are recommented for Figures 1 and 2, and a small additional experiment is recommended for Figure 1:

 Figure 1:

- Because of mobility of imaged structures within the cell, it is difficult to fully ascertain whether blurring and deconvolution of low-intensity fluorescence results in the appearance of biologically relevant structures or artificial creation of structures from background noise. This issue could in principle be resolved by: 1. repeating the experiment in Figure 1 with fixed cells in which the structures are immobile, allowing more direct comparison between visualized structures with 8 scans vs. 1 scan; or (to avoid potential cell fixation artifacts introduced by the above approach) 2. a high-abundance protein that colocalizes with the dim structures could be fluorescently labeled in a second channel and co-visualized (alternatively, a second copy of the same protein could be fused to a bright fluor). This would help validate the expection that the blurred, deconvolved weak signal co-localizes with a stronger marker for the same structure.
- Blurred, pre-deconvolution intermediate images should be shown for both the high-intensity (8 scans) and low-intensity (1 scan) images.

Figure 2:

- Blurred, pre-deconvolution intermediate images should be shown as indicated for Figure 1.

We have read this submission. We believe that we have an appropriate level of expertise to confirm that it is of an acceptable scientific standard.

# Response

**Published:** 2017-08-02

**Authors:** Benjamin Glick

**Affiliations:** University of Chicago, USA

Thanks to the referees for their thoughtful feedback. Please also see the Notes to Readers for an explanation of how we approached this revision.

1) The radius (sigma) value for the Gaussian blur was optimized empirically. We found that a radius of 0.75 – 1.00 pixels worked well for a variety of real and simulated confocal images. This point has been clarified in the text.

2) The pixel size in our images was 80 nm, which is below the resolution limit of the confocal system (~200 nm). With stronger signals, the photons from a fluorescent point source would be spread over multiple pixels. Therefore, our Gaussian blur prefilter generates patterns akin to those seen with well-sampled images.

3) We find that the best way to determine if a structure is biologically relevant is to ask whether it can be tracked between successive frames in a video. By this criterion, the videos associated with the paper confirm that most of the structures seen in the blurred and deconvolved images are indeed biologically relevant.

4) The blurred raw images are actually not very informative, and an extra panel would disrupt the 1:1 correspondence between the figures and the videos. Therefore, unless the referee feels strongly that the blurred images are essential, we would prefer to omit them.

# Peer review: Referee response for version 1

**DOI:** 10.5256/f1000research.12718.r23574

**Published:** 2017-06-19

**Authors:** Akihiko Nakano, Daisuke Miyashiro

**Affiliations:** 1 RIKEN Center for Advanced Photonics, Wako, Japan

This paper describes a tip on using the commercial software Huygens for fluorescent image processing. The authors show that a Gaussian prefilter is useful for preserving very weak signals when the data are deconvolved by Huygens, whose algorithm cannot adequately deal with such images. From a strictly scientific point of view, we think that options of image processing including the use of filters should be discussed for general algorithms not on particular commercial software packages, because they often contain hidden algorithms. Indeed, Huygens’s algorithms are not completely open. However, for most of the cell biologists who are not professional in mathematics, the use of a commercial software package for data processing is common and a tip on obtaining reasonably good-looking data may be helpful for Huygens users.

 We have the following comments.

  

1. The deconvolution method needs be defined more clearly. Making arguments on algorithms with a black box is problematic. Can the details of maximum likelihood algorithm be disclosed, for example, by referring to a document about the method employed by Huygens? How the parameter setting was determined should also be explained.
2. To test the effects of Gaussian blurring on deconvolution of weak signals, the authors performed simulations with a generated set of fluorescent points and PSF. We agree the signal to noise ratio is very important here. In reality, the essential difference between very small numbers of measured photons and background noise must be carefully assessed. Can the authors confirm whether similar conditions are realized on actual conditions under a microscope?
3. The consideration on the unfavorable influence of the Gaussian prefilter is insufficient. The authors suggest using the filter routinely because it gives only a minor blurring effect on strong signals. This is too qualitative. Limitations of the range the method permits should be clearly stated. It will also depend on the purpose of measurements, whether quantitative numerical analysis is required with precision such as particle tracking and edge detection, or qualitative analysis is sufficient such as description of organelle localization dynamics.

 

 Others are minor comments.

  

1. ER-decon depends on a totally different methodology and its comparison is not relevant in this paper.
2. Although the Gaussian blur prefilter has a good characteristic, the rounding error may still cause an unexpected influence on the deconvolution algorithm when the Gaussian radius is not large enough compared to the voxel size. The authors must be aware of this and may want to mention it.

We have read this submission. We believe that we have an appropriate level of expertise to confirm that it is of an acceptable scientific standard, however we have significant reservations, as outlined above.

# Response

**Published:** 2017-08-02

**Authors:** Benjamin Glick

**Affiliations:** University of Chicago, USA

Thanks to the referees for their thoughtful feedback. Please also see the Notes to Readers for an explanation of how we approached this revision.

1) The SVI website provides considerable information about their deconvolution algorithms, but Huygens is necessarily a black box to some degree. Most of the deconvolution parameter settings that we used were either automatic or determined by the properties of the imaging system. The SNR value is crucial, and was chosen empirically for each data set as described  in the Methods.

2) The simulated data were chosen to give results similar to those we obtain from imaging yeast organelles by confocal microscopy. Noise levels in Figure 3B are actually higher than the levels we typically observe.

3) Apart from minor blurring of the final deconvolved structures, we have not noticed ill effects of a Gaussian blur prefilter under any imaging conditions. We have endeavored to emphasize this point in the text. As documented in the paper, our method preserves quantitative information about fluorescence intensities.

4) ER-Decon uses a different algorithm, but like Huygens with a Gaussian blur prefilter, it serves the purpose of deconvolving very weak fluorescence signals. The ER-Decon paper was the first to address this topic in depth and is therefore relevant for our discussion.

5) Empirically, we find that a radius of 0.75 – 1.00 pixel in the Gaussian blur prefilter yields good results. This point is now emphasized more clearly in the text. Note that by using 16-bit data, we avoid the rounding errors that might occur when working with smaller integer values
